# Early School Medicaid Expansions and Health Services for Children With Parental Opioid Use Disorder

**DOI:** 10.1001/jamahealthforum.2025.1288

**Published:** 2025-06-13

**Authors:** Angélica Meinhofer, Lindsey Rose Bullinger, Caroline Hope Kelly, Maria Fitzpatrick

**Affiliations:** 1 Department of Population Health Sciences, Weill Cornell Medicine, New York, New York; 2 School of Public Policy, Georgia Institute of Technology, Atlanta; 3 Crown Family School of Social Work, Policy, and Practice, University of Chicago, Chicago, Illinois; 4 Jeb E. Brooks School of Public Policy, Cornell University, Ithaca, New York

## Abstract

**Importance:**

Children experiencing parental opioid use disorder are a growing population at heightened risk of physical and mental health issues over the life course. Yet these children are less likely to receive comprehensive, ongoing health care and their parents are more likely to report barriers to access health care for their children. School-based health services have potential to overcome some of these health care access barriers, including parental burden, transportation, time, costs, and health care discontinuity. In 2014, Medicaid revoked its longstanding free care rule, expanding the scope of school-based health services eligible for Medicaid reimbursement. Subsequently, some states began to expand their school Medicaid programs to benefit from the new federal rule.

**Objective:**

To estimate the early effects of state school Medicaid expansions on the receipt of Medicaid-funded school-based health services among children who have experienced parental opioid use disorder.

**Design, Setting, and Participants:**

This cohort study using nationwide Medicaid claims data included Medicaid-enrolled children aged 5 to 18 years who experienced parental opioid use disorder at any point before age 19 years. A difference-in-differences design that exploits the staggered implementation of school Medicaid expansions between 2014 and 2019 was used. Data were analyzed between January 2023 and January 2025.

**Exposures:**

Children living in states implementing (treatment group) and not implementing (comparison group) school Medicaid expansions, before and after state-specific expansion dates.

**Main Outcomes and Measures:**

Binary measures indicating receipt of school-based health services, primary care, prevention, rehabilitative, dental, and mental health services, emergency department visits, and inpatient hospital stays.

**Results:**

The sample comprised 6 628 404 person-years from 1 700 304 children. The mean (SD) age was 10.5 (3.9) years and 3 371 918 (51%) were male. School Medicaid expansions increased the receipt of Medicaid-funded school-based health services by 8.9 percentage points (pp; *P* = .01). Growth was primarily driven by school claims for nursing services (difference, 7.4 pp; *P* = .02) and for Early and Periodic Screening, Diagnostic and Treatment services (difference, 8.6 pp; *P* = .04). Reductions in emergency department visits among children aged 5 to 11 years were also documented (difference, −1.8 pp; *P* = .02).

**Conclusions and Relevance:**

This cohort study found that, given the complex health and health care needs of children growing up amid the opioid crisis, integrating health care into schools may offer a promising policy solution.

## Key Points

**Question:**

What is the early impact of state school Medicaid expansions on the receipt of Medicaid-funded school-based health services among children who ever experienced parental opioid use disorder?

**Findings:**

In this cohort study including 6.6 million person-years from 1.7 million children, early state school Medicaid expansions were associated with an 8.9–percentage point increase in the receipt of Medicaid-funded school-based health services. This growth was primarily driven by school claims for nursing services and for Early and Periodic Screening, Diagnostic and Treatment services.

**Meaning:**

Given the complex health and health care needs of children growing up amid the opioid crisis, integrating health care into schools may offer a promising policy solution.

## Teaser

This cohort study estimates the early effects of state school Medicaid expansions on the receipt of Medicaid-funded school-based health services among children who have experienced parental opioid use disorder.

## Introduction

An estimated 2.2 million US children are impacted by opioid use disorder (OUD). Among these children, about 1.5 million live with a parent with OUD, 200 000 have lost a parent to opioid overdose, and more than 300 000 were removed from their home due to parental OUD.^1^ Between 2002 and 2017, the number of children living with adults who had OUD increased by 30%.^2^ In the absence of policy responses, an estimated 4.3 million children will experience parental OUD by 2030.^1^

Parental OUD may lead to a child experiencing unstable or harmful home environments and other adverse childhood experiences, including maltreatment, parental overdose mortality, parental incarceration, and foster care placement.^3^ Children who experienced parental OUD and other adverse childhood experiences are also at heightened risk for a range of physical, mental, and developmental issues over the life course (eg, attention-deficit/hyperactivity disorder, disruptive behaviors, depression, injuries, infections).^4,5,6,7^ Despite their greater health risks, these children are less likely to receive comprehensive, ongoing health care and their parents are more likely to report barriers to access health care for their children.^8^

School-based health services (SBHS) have potential to meet some of the complex health care needs of children experiencing parental OUD through the delivery of primary care, prevention, rehabilitative, and mental health services. Schools are a convenient point of health care access because children are in school for most of the year.^9^ SBHS can therefore reduce parental burden and overcome health care access barriers, including transportation, time, costs, neglect, and health care discontinuity.^10,11^

States often require schools to offer certain SBHS to students free of charge (eg, school nurse).^12^ The costs of SBHS are covered by different funding streams, including federal, state, and local sources of education funding, while Medicaid can reimburse a portion of these costs in select circumstances. Medicaid covers most SBHS through the Early and Periodic Screening, Diagnostic and Treatment (EPSDT) program or through state plan authority.^13^ Prior to 2014, the US Centers for Medicare and Medicaid Services (CMS) prohibited Medicaid reimbursement for SBHS that were available to all students free of charge (known as the free care rule). An exception to this rule was made for Medicaid-enrolled children with Individuals With Disabilities Education Act eligibility but only for SBHS listed in the child’s individualized education plan (IEP). The free care rule was reversed on 2014, when CMS began allowing Medicaid reimbursement for coverable SBHS delivered to all Medicaid-enrolled children by qualified Medicaid-enrolled providers, regardless of whether those SBHS were available to all students free of charge.^14^ As of 2023, 25 states expanded Medicaid reimbursement for SBHS to benefit from the new rule.^15^

While the effects of the opioid crisis on adults and newborns have been widely studied, its impact on school-aged children—and the role of schools and other support systems in addressing their needs—remains understudied. We used nationwide Medicaid claims and a difference-in-differences (DD) design to generate the first estimates of the early effects of school Medicaid expansions on the receipt of Medicaid-funded SBHS and other health services among children who ever experienced parental OUD.

The large number of children growing up amid the opioid crisis demands novel health care models for mitigating the intergenerational effects of parental OUD. School Medicaid expansions could be crucial for these children, who often experience neglect, are enrolled in Medicaid, have complex and unmet health care needs, and may benefit from convenient health care access that eases parental burden.

## Methods

### Data

We analyzed Medicaid claims from the 2014-2015 Medicaid Analytical eXtract (MAX) and 2014-2020 Transformed Medicaid Statistical Information System Analytic Files. Data are nationally representative, capturing eligibility and claims records for all Medicaid and Children’s Health Insurance Program enrollees in the 50 states, Puerto Rico, and the District of Columbia. Data include demographic and eligibility files, inpatient claims, other services claims, and prescription drug pharmacy claims. The Beneficiary ID identifies a beneficiary over time and across states. The state-assigned CaseID identifies families in most states.^16,17^ The Weill Cornell Medicine Institutional Review Board deemed this study exempt and waived the informed consent requirement because secondary data excluded direct identifiers. This study followed the Strengthening the Reporting of Observational Studies in Epidemiology (STROBE) reporting guideline.

### Study Population

We selected school-aged children (aged 5 to 18 years) who experienced parental OUD if, at any point before age 19 years, in their claims records: (1) diagnostic codes indicated perinatal opioid exposure or drug addiction in the family; (2) CaseID linked them to a likely sibling (younger than 19 years) with an age gap of 10 years or less and who experienced parental OUD; (3) CaseID linked them to a likely parent (aged 19 to 64 years) with an age gap of 18 years or more and OUD, identified with diagnostic codes indicating opioid abuse, dependence, or poisoning or with a procedure or national drug code indicating OUD treatment with methadone or buprenorphine. The latter 2 selection criteria leveraged the claims records of family members, linked to the child with a family linkage algorithm requiring the same CaseID and zip code or county of residence (eMethods 1 in Supplement 1).^16,17,18^ We excluded inaccurate linkages and dropped beneficiaries with missing CaseID, Beneficiary ID , or birth date. We imposed eligibility and enrollment criteria to increase claim record completeness. We excluded person-years with less than 90 days of comprehensive enrollment, missing eligibility information, with State Children’s Health Insurance Program enrollment, dual Medicare and Medicaid insurance, and restricted Medicaid benefits (eMethods 1 in Supplement 1). We dropped 2020 due to the COVID-19 pandemic and states with poor reporting (New York, California, Utah, and the Virgin Islands; eMethods 1 in Supplement 1).

We structured the data as a longitudinal sample of person-years, where each child could be observed for up to 6 years between 2014 and 2019. eFigure 1 in Supplement 1 plots raw time trends for population characteristics.

### Outcome Variables

Outcomes included Medicaid-funded SBHS and other health services directly or indirectly affected by school Medicaid expansions. We generated indicators identifying person-years in which a child had at least 1 claim involving a given service. We identified services in inpatient and outpatient claims using procedure codes and other data elements (eMethods 2 in Supplement 1 and the eTable in Supplement 2). We prioritized procedure codes from validation studies when available and from school Medicaid program billing guides and technical reports.

We identified SBHS claims following a CMS algorithm,^19^ which uses a combination of data elements, including place of service, provider taxonomy, benefit type, service type, funding source, and procedure codes (eTable 2 in Supplement 1). Medicaid does not require states to follow a standard method for reporting SBHS claims, which created challenges for separately identifying SBHS types. Put differently, a same SBHS claim may be recorded under some data elements but not others depending on the state, and these elements vary in their level of detail. We therefore implemented an imputation approach and generated several SBHS measures (eMethods 2 in Supplement 1): (1) school settings, capturing IEP and non-IEP services delivered in school settings by school providers or providers working independently; (2) school providers, capturing IEP and non-IEP services where the billing or servicing provider was Medicaid enrolled and used school-related taxonomy codes; (3) IEP, capturing IEP services delivered in school and nonschool settings (eg, transportation from school to another health care setting), by school providers or providers working independently; (4) any SBHS, combining measures 1 to 3. We excluded state-years in which a given imputed SBHS measure was missing for 30% or more of beneficiaries.

We identified other health services generally provided in the EPSDT benefit (eg, screening, diagnostics, treatment), IEP (eg, rehabilitation), or schools (eg, nursing). Specific outcomes included EPSDT-financed claims, well-child visits, nursing services, dental services, mental health services, hearing/vision examinations, rehabilitation services (eg, physical therapy, occupational therapy, and hearing and speech language services), and nonemergency medical transportation. We classified these services by SBHS claims and any claims to examine potential substitution of health services previously delivered in nonschool settings. Lastly, we analyzed inpatient hospital stays and emergency department visits to assess whether school-based prevention and primary care services help reduce the need for these intensive and costly services.

### School Medicaid Expansions

The free care rule was reversed on December 15, 2014.^12^ Subsequently, select states began to expand their school Medicaid programs to benefit from the new rule (eTable 3 in Supplement 1). There was considerable state variation in the scope of newly eligible services, students, and providers. Between 2014 and 2019, 12 states expanded school Medicaid (eMethods 3 in Supplement 1). We used state-specific effective dates of school Medicaid expansions collected by the Healthy Schools campaign.^13^ Additionally, we interviewed officials from several school Medicaid programs and read relevant documentation in state websites and other official sources to confirm some dates. This definition included states expanding Medicaid reimbursement for SBHS beyond an IEP through (1) state plan amendment using the CMS approval date or subsequent policy date in the handful of states where additional legislation was needed for expansion or (2) state legislation or administrative action without state plan amendment using the implementation date. Interviews with state officials revealed that school districts within expanding states varied in their decisions to expand school Medicaid and experienced delays during the expansion process. We therefore expect that policy effects, if any, would be delayed.

### Statistical Analysis

We estimated DD models with the two-way fixed-effects estimator, which compares states that expanded school Medicaid (treatment group) and states that did not (comparison group) before and after state-specific effective dates of expansion (eMethods 4 in Supplement 1).

The binary outcome equaled 1 if a child had at least 1 claim involving a given service in the person-year and 0 otherwise. The policy variable equaled 1 if the state expanded school Medicaid during the full year and 0 otherwise. To minimize measurement error, this variable equaled the proportion of treated months in the effective year. Regressions controlled for zip code fixed effects, year fixed effects, indicator of Affordable Care Act Medicaid expansions, indicator of Medicaid Analytical eXtract state-years, and individual-level covariates (race and ethnicity, sex, age, annual enrollment days).^20^ We estimated linear probability regressions and state-level clustered standard errors. Additionally, we estimated event study regressions to evaluate if treatment effects were dynamic and whether the parallel trends assumption required for DD models appeared reasonable.

Lastly, we conducted various sensitivity checks, including dropping controls; analyzing nonimputed SBHS measures; excluding states where data element(s) used to generate a given SBHS measure were present but never indicated school-related codes, despite other data elements indicating school-related codes; applying state fixed effects; applying person fixed effects; using the multiperiod DD estimator, which is robust to bias from treatment effect heterogeneity across states and over time^21,22^; excluding states with low CaseID linkage rates; excluding Washington, which expanded school Medicaid through Managed Care; and modifying selection criteria related to parental OUD exposure.

Two-tailed *P* values were calculated using *t* tests, and significance was set at *P* < .05. All analyses were conducted using Stata version 18 (StataCorp).

## Results

The analytical sample comprised 6 628 404 person-years from 1 700 304 Medicaid-enrolled children who experienced parental OUD. The mean (SD) age was 10.5 (3.9) years and 3 371 918 (51%) were male (eTable 1 in Supplement 1). The mean (SD) number of years observed was 4 (1.9) years and of annual enrollment days was 340 (66.3) days.

**Figure 1.**
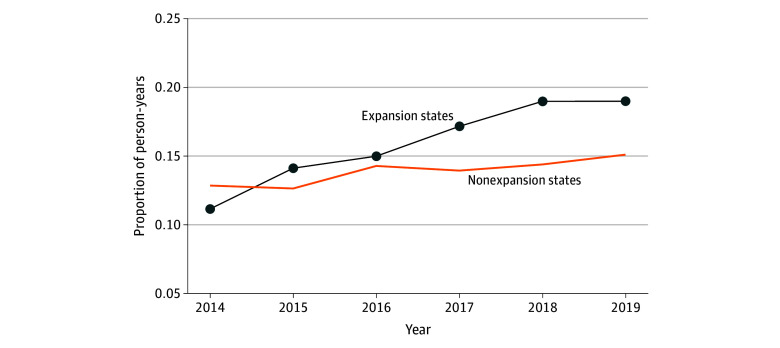
Trends in Medicaid-Funded School-Based Health Services by School Medicaid Expansion Status The unit of analysis are person-years (n = 6 628 404) from 1 700 304 children aged 5 to 18 years experiencing parental opioid use disorder. The figure captures time trends in the raw proportion of children with at least 1 Medicaid claim for any school-based health services in the year, stratified by school Medicaid expansion status. States expanding their school Medicaid programs by December 31, 2019, were assigned to the expansion states group. National Medicaid claims data are from the 2014-2019 Transformed Medicaid Statistical Information System Analytic Files and the 2014-2015 Medicaid Analytical eXtract.

**Figure 2.**
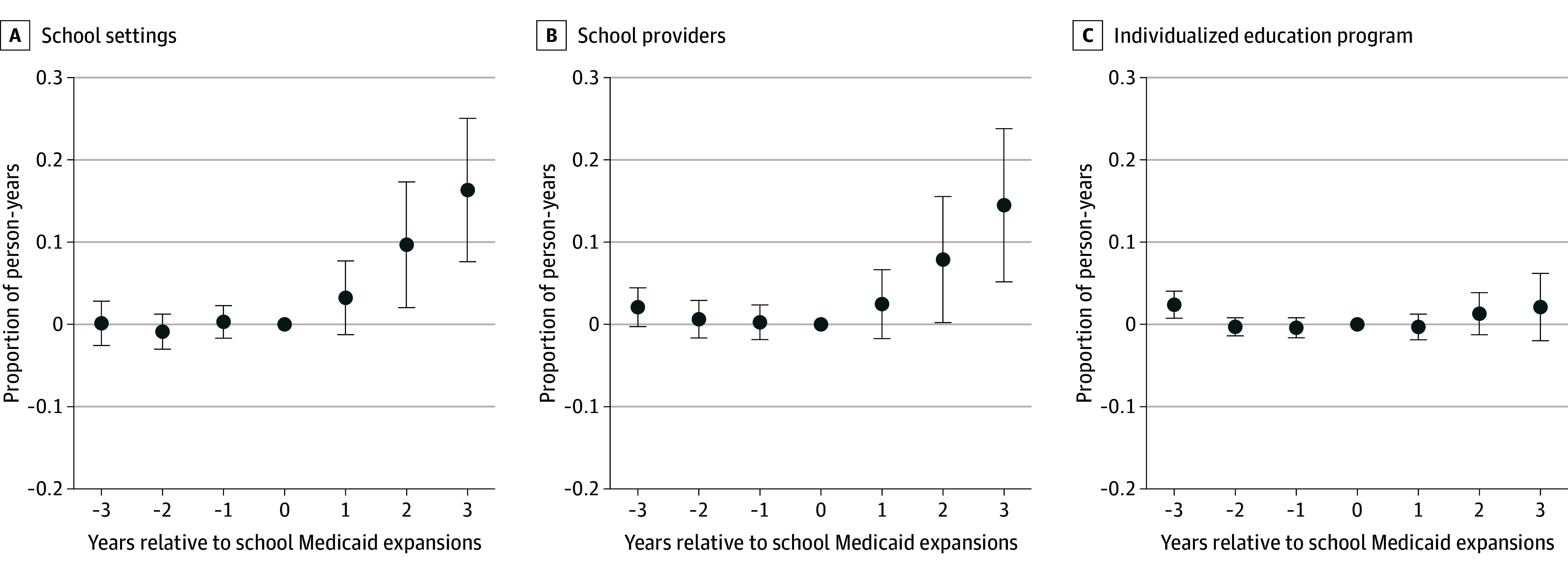
Event Study Plots of School Medicaid Expansions and School-Based Health Services The unit of analysis are person-years (n = 6 628 404) from 1 700 304 children aged 5 to 18 years experiencing parental opioid use-related disorder. Coefficients are based on event study regressions that control for leads and lags of the policy, along with child demographic characteristics, Medicaid enrollment days, zip code fixed effects, year fixed effects, Medicaid Analytical eXtract state-years, and Affordable Care Act Medicaid Expansions. The 95% CIs are clustered at the state level. The reference year is time 0, the year immediately before the implementation of school Medicaid expansions. National Medicaid claims data are from the 2014-2019 Transformed Medicaid Statistical Information System Analytic Files and the 2014-2015 Medicaid Analytical eXtract. Error bars indicate 95% CIs.

Figure 1 plots trends in the raw proportion of children receiving any SBHS by school expansion status. Between 2014 and 2019, SBHS receipt increased faster in expansion states (from 0.111 to 0.189, a 70% increase) relative to nonexpansion states (from 0.128 to 0.151, an 18% increase). Figure 2 plots event study regressions of the association of school Medicaid expansions with SBHS receipt. Expansion and nonexpansion states trended similarly during the years leading up to school Medicaid expansions. Following implementation, SBHS in school settings or by school providers increased over time in expansion states. IEP services, however, remained unchanged.

**Figure 3.**
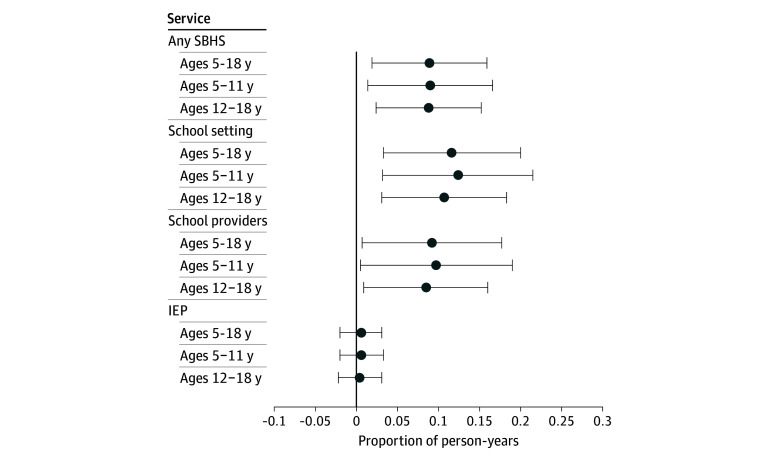
Association of State School Medicaid Expansions With School-Based Health Services (SBHS) The unit of analysis are person-years (n = 6 628 404) from 1 700 304 children aged 5 to 18 years experiencing parental opioid use disorder. Coefficients are based on difference-in-differences regressions that control for zip code fixed effects, year fixed effects, Medicaid Analytical eXtract state-years, child demographic characteristics, Medicaid enrollment days, and Affordable Care Act Medicaid Expansions. The 95% CIs are clustered at the state level. Full regression output is reported in eMethods 4 in Supplement 1. National Medicaid claims data are from the 2014-2019 Transformed Medicaid Statistical Information System Analytic Files and the 2014-2015 Medicaid Analytical eXtract. Error bars indicate 95% CIs. IEP indicates individualized education plan.

Figure 3 reports DD point estimates. School Medicaid expansions significantly increased the proportion of children receiving SBHS by school providers (difference, 9.2 percentage points [pp]; *P* = .03) and in school settings (difference, 11.6 pp; *P* = .007). We observed no statistically significant differences in IEP services. Together, SBHS grew by 8.9 pp (*P* = .01), an 81% increase relative to the baseline mean in expansion states.

**Figure 4.**
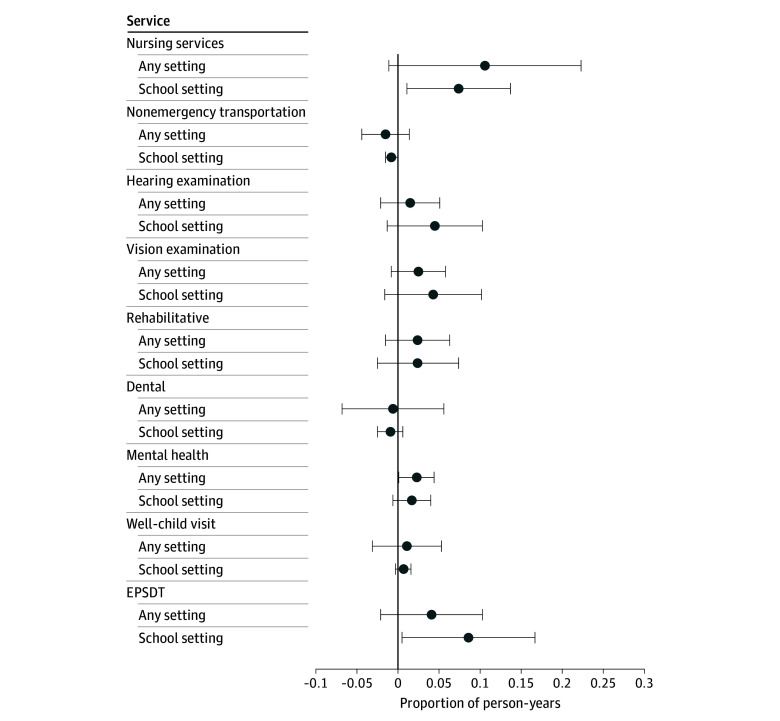
Association of State School Medicaid Expansions With Health Services by School Setting The unit of analysis are person-years (n = 6 628 404) from 1 700 304 children aged 5 to 18 years experiencing parental opioid use disorder. Coefficients are based on difference-in-differences regressions that control for zip code fixed effects, year fixed effects, Medicaid Analytical eXtract state-years, child demographic characteristics, Medicaid enrollment days, and Affordable Care Act Medicaid Expansions. The 95% CIs are clustered at the state level. Full regression output is reported in eMethods 4 in Supplement 1. National Medicaid claims data are from the 2014-2019 Transformed Medicaid Statistical Information System Analytic Files and the 2014-2015 Medicaid Analytical eXtract. Error bars indicate 95% CIs. EPSDT indicates Early and Periodic Screening, Diagnostic and Treatment.

Figure 4 reports DD estimates for specific health services by SBHS claims and any claims. Growth in SBHS claims was driven by EPSDT (difference, 8.6 pp; *P* = .04) and nursing (difference, 7.4 pp; *P* = .02) services. We also observed reductions in nonemergency medical transportation in schools (difference, 0.8 pp; *P* = .045). We found null associations with other health services in schools, although point estimates for mental health services, hearing examinations, vision examinations, and rehabilitative services were positive and sizable relative to respective baseline means. When considering any claims, regardless of SBHS, we documented net increases in mental health services (difference, 2.3 pp; *P* = .04) and nursing services (difference, 10.6 pp; *P* = .07).

**Figure 5.**
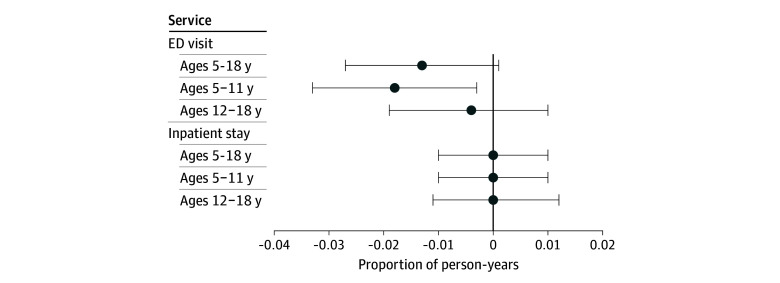
Association of State School Medicaid Expansions With Hospital Services The unit of analysis are person-years (n = 6 628 404) from 1 700 304 children aged 5 to 18 years experiencing parental opioid use disorder. Coefficients are based on difference-in-differences regressions that control for zip code fixed effects, year fixed effects, Medicaid Analytical eXtract state-years, child demographic characteristics, Medicaid enrollment days, and Affordable Care Act Medicaid Expansions. The 95% CIs are clustered at the state level. Full regression output is reported in eMethods 4 in Supplement 1. National Medicaid claims data are from the 2014-2019 Transformed Medicaid Statistical Information System Analytic Files and the 2014-2015 Medicaid Analytical eXtract. Error bars indicate 95% CIs. ED indicates emergency department.

We next inquired whether SBHS growth subsequently reduced health services in intensive and costly settings. Figure 5 shows modest reductions in the proportion of emergency department visits but only among children aged 5 to 11 years (difference, 1.8 pp; *P* = .02). We did not find statistically significant effects on inpatient stays.

Regression output for main estimates and sensitivity analyses can be found in eMethods 4 and 5 and eTables 4 to 8 in Supplement 1. Estimates were robust to sensitivity checks.

## Discussion

The free care rule reversal could help meet the health care needs of many underserved children with complex health conditions, including the large and fast-growing population of children who experienced parental OUD. We generated the first estimates of the early effects of school Medicaid expansions on the receipt of Medicaid-funded SBHS in this population and documented several findings.

First, we found considerable, albeit delayed, increases in SBHS receipt following school Medicaid expansions. SBHS delivered within school settings and by school providers accounted for most of this growth. There were insignificant effects on IEP services, which is expected since these were already covered by Medicaid pre-2014 and therefore likely unaffected by the rule reversal and subsequent state expansions. A related report documented null differences in Medicaid SBHS expenditures between states with high vs low SBHS reimbursement barriers.^23^ These null findings likely result from assigning 2014 as the expansion date for all treated states rather than using state-specific expansion dates. Additionally, SBHS expenditures captured school-based administration and school-based services as defined in the CMS-64 form.^24^ The former includes administrative activities not associated with medical services and unaffected by the free care rule (eg, Medicaid outreach, eligibility determinations, general administration).^21^ The latter does not reflect all SBHS and likely primarily reflects IEP services.^21,25^ Therefore, the report’s null findings are consistent with our null IEP findings. Together, our documented increases in SBHS by school providers and settings, along with null IEP effects align with provisions of the rule reversal, strengthening the credibility and interpretation of our findings. Delayed SBHS increases align with reported implementation delays among expanding states.

Second, SBHS growth was primarily driven by EPSDT and nursing services. Many states or districts mandate schools to offer select health services free of charge to all students, financed by education funds. These often include services delivered by school nurses, psychologists, and social workers, such as medication monitoring or select EPSDT services.^12^ As services offered free of charge to all students were not reimbursable by Medicaid under the free care rule, it is not surprising that it is precisely these services where claims increased most following school Medicaid expansions, at least in the short term. It is possible that expansions will induce schools to broaden the scope of SBHS offered in the long term. Notably, we observed modest reductions in nonemergency medical transportation in school settings, which suggests schools are better equipped to provide health services in-house rather than having to relocate children.

Third, the magnitude of the point estimate for SBHS claims involving nursing services was similar to or slightly smaller than that for any claims involving nursing services. This suggests that Medicaid-funded nursing services delivered in school settings did not crowd out same services delivered in nonschool settings. However, it does not rule out crowd out of nursing services previously and currently delivered in school settings but previously financed by non-Medicaid sources (eg, education funds) before school expansions. In contrast, EPSDT point estimates suggest that about one-half of the new EPSDT receipt in schools was previously delivered in nonschool settings.

Finally, we found modest decreases in emergency department visits among children aged 5 to 11 years. Emergency department overutilization is a well-documented issue in the US health care system, contributing to costs and fragmented care.^26^ Nonemergency primary care sought in the emergency department largely results from individuals who cannot easily access health care; this is especially true for low-income children.^27,28,29,30^ By expanding coverage of health services in schools, a convenient setting, school Medicaid expansions might prevent the overutilization of these intensive and costly health services.

Our results have implications for the policy landscape regarding health care in schools. At the federal level, the 2022 Bipartisan Safer Communities Act included funding for expanding school-based mental health services and providing technical assistance for state Medicaid agencies and school districts. At the state level, nearly one-half of US states have yet to expand their school Medicaid programs, and many expanding states only cover a limited set of services, providers, or children. Our findings imply that these policies represent an opportunity to offer a sustainable funding source to provide health services in schools and will serve to increase health care access among children experiencing parental OUD and potentially other underserved children.

### Limitations

This study has limitations. First, characterizing complex state policies with a binary indicator and effective dates likely misses nuanced differences in scope and timing of implementation. Second, Medicaid claims do not identify familial relationships between beneficiaries sharing a CaseID, and therefore our approximation using age may not always correctly identify parents or siblings. Third, Medicaid claims do not capture services funded by other sources, such as education funds. Therefore, outcomes may not reflect net health care receipt. Fourth, Medicaid claims may underreport OUD and parental OUD prevalence and duration. Fifth, some SBHS variables are inconsistently reported in select state-years. Still, estimates are robust to different SBHS definitions and imputation approaches. Sixth, we cannot rule out compositional bias from beneficiaries entering and exiting the sample, which could arise since we do not use a continuously enrolled sample or if school Medicaid expansions themselves influence Medicaid enrollment. Nevertheless, estimates are robust to person fixed effects, which use within-person variation and are less affected by changes in sample composition. Lastly, estimates reflect short-term effects of school Medicaid expansions.

## Conclusions

School Medicaid expansions could transform health care access for Medicaid-enrolled children with parental OUD. We showed that the receipt of Medicaid-funded SBHS increased following expansions, although it remains unclear whether this reflects a shift in funding source (from education funds to Medicaid reimbursement) or a net increase in health care utilization. Our documented declines in emergency department visits suggest that increased health care utilization or quality—not just a funding source shift—may be at play to some extent. At a minimum, our findings demonstrate that in the short term, more children received Medicaid-funded nursing and EPSDT services in schools, boosting Medicaid reimbursement for schools and facilitating earlier detection and management of health conditions that might otherwise lead to emergency care. Long term, these funding shifts and greater care proximity will likely enhance health care quality and availability of additional SBHS, leading to improved child health and well-being—a trend future research may confirm. Given the pressing health care challenges facing children who have experienced parental OUD, integrating health care into schools presents a promising policy solution.

## Notes

Supplement 1. **eMethods 1.** Study Population **eMethods 2.** Study Measures **eTable 1.** Summary Statistics, 2014-2019 **eFigure.** Time Trends in Study Measures **eTable 2.** School-Based Health Services Claims **eMethods 3.** Effective Dates of School Medicaid Expansions **eTable 3.** Effective Dates of School Medicaid Expansions as of 2019 **eMethods 4.** Difference-in-Differences Regressions **eTable 4.** Effect of School Medicaid Expansions on School-Based Health Services **eTable 5.** Effect of School Medicaid Expansions on Other Health Services by SBHS Claims **eTable 6.** Effect of School Medicaid Expansions on Hospital Services **eMethods 5.** Sensitivity Analyses **eTable 7.** Effect of School Medicaid Expansions on School-Based Health Services, Robustness Checks **eTable 8.** Effect of School Medicaid Expansions on School-Based Health Services, POUD Exposure

Supplement 2. Procedure Codes and Data Elements Used to Identify Measures

Supplement 3. Data Sharing Statement
