# Supplementary material for: Early School Medicaid Expansions and Health Services for Children With Parental Opioid Use Disorder
Source: JAMA Health Forum. 2025 Jun 13;6(6):e251288. doi: 10.1001/jamahealthforum.2025.1288 (PMC12166484; doi:10.1001/jamahealthforum.2025.1288)
Supplement: Supplement 1. — eMethods 1. Study Population eMethods 2. Study Measures eTable 1. Summary Statistics, 2014-2019 eFigure. Time Trends in Study Measures eTable 2. School-Based Health Services Claims eMethods 3. Effective Dates of School Medicaid Expansions eTable 3. Effective Dates of School Medicaid Expansions as of 2019 eMethods 4. Difference-in-Differences Regressions eTable 4. Effect of School Medicaid Expansions on School-Based Health Services eTable 5. Effect of School Medicaid Expansions on Other Health Services by SBHS Claims eTable 6. Effect of School Medicaid Expansions on Hospital Services eMethods 5. Sensitivity Analyses eTable 7. Effect of School Medicaid Expansions on School-Based Health Services, Robustness Checks eTable 8. Effect of School Medicaid Expansions on School-Based Health Services, POUD Exposure [file jamahealthforum-e251288-s001.pdf]

Supplemental Online Content

Meinhofer A, Bullinger LR, Kelly CH, Fitzpatrick M. Early school Medicaid expansions and health services for children with parental opioid use disorder. *JAMA Health Forum.* 2025;6(6):e251288. doi:10.1001/jamahealthforum.2025.1288

eMethods 1. Study Population
eMethods 2. Study Measures
eTable 1. Summary Statistics, 2014-2019
eFigure. Time Trends in Study Measures
eTable 2. School-Based Health Services Claims
eMethods 3. Effective Dates of School Medicaid Expansions
eTable 3. Effective Dates of School Medicaid Expansions as of 2019
eMethods 4. Difference-in-Differences Regressions
eTable 4. Effect of School Medicaid Expansions on School-Based Health Services
eTable 5. Effect of School Medicaid Expansions on Other Health Services by SBHS Claims
eTable 6. Effect of School Medicaid Expansions on Hospital Services
eMethods 5. Sensitivity Analyses
eTable 7. Effect of School Medicaid Expansions on School-Based Health Services, Robustness Checks
eTable 8. Effect of School Medicaid Expansions on School-Based Health Services, POUD Exposure

This supplementary material has been provided by the authors to give readers additional information about their work.

© 2025 Meinhofer A et al. *JAMA Health Forum*.

eMethods 1. Study Population

We describe the sample selection criteria below, which follows a similar approach as that described in previous studies. We identified school-aged children ages 5-18 who experienced POUD if at any point between the ages 0-18, their claims records: (1) directly indicated perinatal opioid exposure or drug addiction in the family; (2) linked them to a likely sibling ages 0-18 with POUD exposure as defined in (1) and age gap of 10 years or less; (3) linked them to a likely parent ages 19-64 diagnosed with OUD and age gap of 18 years or more. Since we allow POUD exposure to occur at any point during childhood and use multiple selection criteria, there will be heterogeneity among children in terms of the nature and ongoing status of their POUD exposure at the time of their healthcare utilization. Examples of this heterogeneity can include differences in whether the parents are actively using substances; are receiving OUD treatment; are in recovery; have custody of the child; or in the timing and duration of these exposures. Despite this heterogeneity, our broader approach ensures that we capture children affected by both the immediate and long-lasting consequences of POUD and consider their full range of needs. This is crucial because POUD is an adverse childhood experience (ACE) that can be both preceded by and contribute to other vulnerabilities, leading to unstable or harmful home environments, neglect, and the accumulation of additional ACEs. These effects of POUD are both immediate and long-lasting, influencing children's well-being beyond the period of active parental substance use. Given the enduring impact of POUD, it is critical to examine how school Medicaid expansions and school-based health services (SBHS) can support children who have *ever* experienced POUD, not just those currently facing it.

© 2025 Meinhofer A et al. *JAMA Health Forum*.

eMethods A.1. Selection Criteria (1)

To identify children who experienced POUD meeting selection criteria (1), we used diagnostic codes in their inpatient and other services claims records indicating perinatal exposure to opioids (i.e. neonatal drug withdrawal syndrome) or drug addiction in the family. We then used birth date to impose that these diagnostic codes were observed at least once between ages 0-18. There were n=382,224 children meeting selection criteria (1).

eMethods A.2. Selection Criteria (2) and (3)

The selection criteria (1) described in A.1 will identify some but not all children who ever experienced POUD. For example, not all children with prenatal exposure to opioids may develop neonatal drug withdrawal syndrome and healthcare providers may not consistently report drug addiction in the family using diagnostic codes. We therefore used the claims records of family members —namely, (2) likely siblings who experienced POUD or (3) likely parents diagnosed with OUD (collectively referenced as O-relatives hereafter)— to identify additional children who experienced POUD. We implemented an established and validated family linkage algorithm (sensitivity varies across states, but positive predictive value exceeds 99%), modified to fit our populations of interest. This algorithm requires the child to share the same Case ID and ZIP or county of residence as the O-relative.

First, we identified O-relatives. We identified potential likely siblings who have experienced POUD as children ages 0-18 meeting selection criteria (1) in A.1. We identified potential likely parents diagnosed with OUD as adults ages 19-64 with diagnostic codes indicating opioid abuse, dependence, or poisoning, or with procedure codes or national drug codes indicating OUD medication treatment with methadone or buprenorphine. Medicaid claims

© 2025 Meinhofer A et al. *JAMA Health Forum*.

will underreport beneficiaries with OUD who lack diagnostic codes for the condition. This underreporting likely occurs among those who do not seek Medicaid-funded healthcare, do not disclose their OUD to providers, or receive care from providers who do not report the diagnosis.

Second, we used all Case IDs ever assigned to these O-relatives to link them with all other beneficiaries sharing the same Case ID at least once during the study period. Third, we dropped Case IDs unlikely to reflect family units, which included: [**A**] Case IDs associated with a single beneficiary. The family linkage rate was initially low in seven states. In three states, we fixed these linkage issues by using the unencrypted Case ID along with the leading number of Case ID digits previously described.[1] In four states (CT, WA, TX, NC), however, the Case ID did not link families in most or all years. Therefore, our sample did not properly capture children under selection criteria (2) and (3) in those states, except for a subset of children who moved to one of those states but was linked to an O-relative while living in a different state or who was observed on select years where the Case ID did link families in those states. This limitation did not affect children who experienced POUD under selection criteria (1) in those states. Therefore CT, WA, TX, and NC were all included in our sample and in robustness checks we confirmed that estimates were not substantively different when these states were excluded (eTable 8). [**B**] Case IDs associated with more than 15 beneficiaries or with more than eight distinct ZIP codes, since a small number of Case IDs matched to an unreasonably high number of beneficiaries or ZIP codes in a few instances. Fourth, we imposed age and age gap requirements between the child and their linked O-relative consistent with either a sibling or parental familial relationship. We selected children ages 0-18 either sharing an age gap of 10 years or less with the likely sibling who experienced POUD, or sharing an age gap of 18 years or more with the likely parent diagnosed with OUD. Fifth, we required the child and O-relative to share the same ZIP or county

of residence at least once during the sample period. There were an additional n=2,581,820 beneficiaries meeting selection criteria (2) or (3).

Together, there were n=2,964,044 beneficiaries meeting selection criteria (1), (2) or (3), out of which 13% met selection criteria (1), 17% met selection criteria (2), and 80% met selection criteria (3). As such, some beneficiaries met multiple selection criteria. If excluding the four states with low Case ID linkage rate (CT, WA, TX, NC), among beneficiaries meeting selection criteria (1), 13% also met selection criteria (2) and 40% also met selection criteria (3). Among beneficiaries meeting selection criteria (2), 38% also met selection criteria (3).

eMethods A.3. Enrollment and Eligibility Criteria

We combined the claims records of all 2,964,044 children identified as experiencing POUD using selection criteria (1), (2) or (3). We then imposed the following continuous enrollment and eligibility criteria following previous studies and definitions in the Centers for Medicare & Medicaid Services TAF Technical Guide: We dropped person-years with less than 90 days of enrollment; without comprehensive benefits for all months of enrollment (e.g., family planning only, or care limited to a specific condition); missing eligibility information for all months of enrollment; enrolled in separate state S-CHIP for all months of enrollment; or with dual health insurance (i.e., Medicare and Medicaid) for all months of enrollment. There were n=2,900,185 beneficiaries meeting this selection criteria.

eMethods A.4. Other Selection Criteria

We dropped the year 2020 due to potential confounding from the COVID-19 pandemic, which resulted in nationwide school closures. We also dropped states or state-years with data quality

© 2025 Meinhofer A et al. *JAMA Health Forum*.

issues. Missingness and other quality issues affecting a particular outcome depend on the data elements used to generate it (e.g. place of service, provider taxonomy, Medicaid provider ID, procedure codes, benefit type, service type), and states might have quality issues affecting a single or multiple data elements in a single or multiple years. Our sample entirely excludes all observations from four states (NY, CA, UT, VI) with a high rate of missing values across multiple data elements and/or across multiple years. We kept person-years in which a child was ages 5 to 18. We structured the data as a longitudinal sample of person-years, where each child could be observed for up to 6 years between 2014-2019. The sample comprised 6,628,404 person-years from 1,700,304 Medicaid-enrolled children who ever experienced POUD during ages 5-18. The first year of POUD exposure documented in Medicaid claims was equal to or preceded 63% of the 6,628,404 person-years. POUD exposure was documented during 2 or more calendar years for 56% of the 1,700,304 children.

© 2025 Meinhofer A et al. *JAMA Health Forum*.

eMethods 2. Study Measures

We generated study measures using diagnostic and procedure codes from the International Classification of Diseases Ninth (ICD-9) and Tenth (ICD-10) Revision, Current Procedural Terminology-4 (CPT), Healthcare Common Procedure Coding System (HCPCS), and Current Dental Terminology (CDT) codes, as well as other data elements in Medicaid claims data. We prioritized codes from validation studies when available, as well as from state Medicaid billing guides and CMS technical reports.

eMethods B.1 Summary Statistics and Descriptive Plots

Summary statistics for outcome and control measures are in eTable 1. Specific procedure codes and data elements used to identify these measures are in the eTable in Supplement 2, included as a separate Excel spreadsheet.

During our study period of 2014-2019, there were two data transitions that could affect study measures and sample selection. First, Medicaid claims data transitioned from MAX ("old generation") to TAF ("new generation"). TAF is designed to better standardize claims data collection across states, add data quality enhancements, provide a more comprehensive set of data elements, and improve usability. Therefore, there are differences in data elements, structure, and quality between MAX and TAF. There is a combination of MAX/TAF states from 2014 to 2015 (26 MAX states in 2014 and 17 MAX states in 2015) and starting in 2016, only TAF states. Second, ICD-9 transitioned to ICD-10 in 2015Q4, improving diagnostic code specificity but also creating mapping challenges in some cases. This represented less of an issue for our study measures since most outcomes relied on CPT and HCPCS procedure codes or data elements other than diagnostic codes, but could affect diagnostic codes of OUD/POUD used for sample selection. To verify the stability of our study measures and the study population during these

transitions, eFigure 1 plots raw time trends for key study measures and population characteristics. As shown, study measures are stable during our study period, including during MAT-TAF years and TAF-only years.

© 2025 Meinhofer A et al. *JAMA Health Forum*.

eTable 1. Summary Statistics, 2014-2019

	Expanded School Medicaid		Control	Total
	Before	After		
Person Years (PY)	878,252	480,919	5,269,233	6,628,404
Demographic Controls				
Female	0.49	0.49	0.49	0.49
Male	0.51	0.51	0.51	0.51
Age (Mean)	10.39	10.49	10.53	10.51
Age 5 to 11	0.62	0.61	0.60	0.61
Age 12 to 18	0.38	0.39	0.40	0.39
Black, NH	0.13	0.17	0.18	0.17
White, NH	0.70	0.69	0.60	0.62
Other Race, NH	0.03	0.02	0.06	0.05
Hispanic	0.06	0.07	0.12	0.11
Missing Race	0.07	0.04	0.05	0.05
Medicaid Controls				
Enrollment days (Mean)	343.49	346.09	339.07	340.16
ACA Medicaid Expansions	0.87	0.80	0.69	0.72
School-Based Health Services				
Any SBHS	0.11	0.26	0.14	0.14
School Setting	0.10	0.26	0.11	0.12
School Provider	0.05	0.19	0.07	0.08
Individualized Education Program	0.04	0.09	0.05	0.05
Other Health Services in School Settings				
Well-Child Visit	0.01	0.01	0.02	0.02
EPSDT	0.01	0.15	0.03	0.04
Nursing Services	0.01	0.12	0.01	0.02
Mental Health Visits	0.03	0.05	0.04	0.04
Dental Services	0.01	0.01	0.03	0.03
Rehabilitative	0.05	0.12	0.08	0.08
Vision Examination	0.00	0.07	0.02	0.02
Hearing Examination	0.01	0.07	0.02	0.02
Non-Emergency Medical Transportation	0.002	0.001	0.015	0.013
Other Health Services in Any Settings				
Well-Child Visit	0.47	0.47	0.44	0.45
EPSDT	0.58	0.64	0.44	0.47
Nursing Services	0.23	0.40	0.18	0.21
Mental Health Visits	0.18	0.22	0.20	0.20
Dental Services	0.56	0.57	0.52	0.53
Rehabilitative	0.23	0.32	0.31	0.30
Vision Examination	0.14	0.23	0.19	0.19
Hearing Examination	0.14	0.22	0.16	0.16
Non-Emergency Medical Transportation	0.02	0.02	0.09	0.08
Hospital Settings				
Inpatient Stays	0.02	0.02	0.02	0.02
Emergency Room Visits	0.33	0.32	0.32	0.32
Physical and Mental Health Outcomes				
Congenital Anomalies	0.02	0.02	0.02	0.02
Complex Chronic Conditions	0.05	0.05	0.05	0.05
Developmental Delays	0.06	0.07	0.07	0.07
Injuries	0.21	0.21	0.18	0.19
Substance Use Disorders	0.01	0.01	0.01	0.01
Depression	0.04	0.05	0.04	0.04
Anxiety	0.05	0.06	0.05	0.05

© 2025 Meinhofer A et al. *JAMA Health Forum*.

Trauma	0.09	0.10	0.08	0.08
ADHD	0.15	0.18	0.13	0.14

Notes:

Statistics reflect the proportion of person-years, unless otherwise indicated.

© 2025 Meinhofer A et al. *JAMA Health Forum*.

eFigure. Time Trends in Study Measures

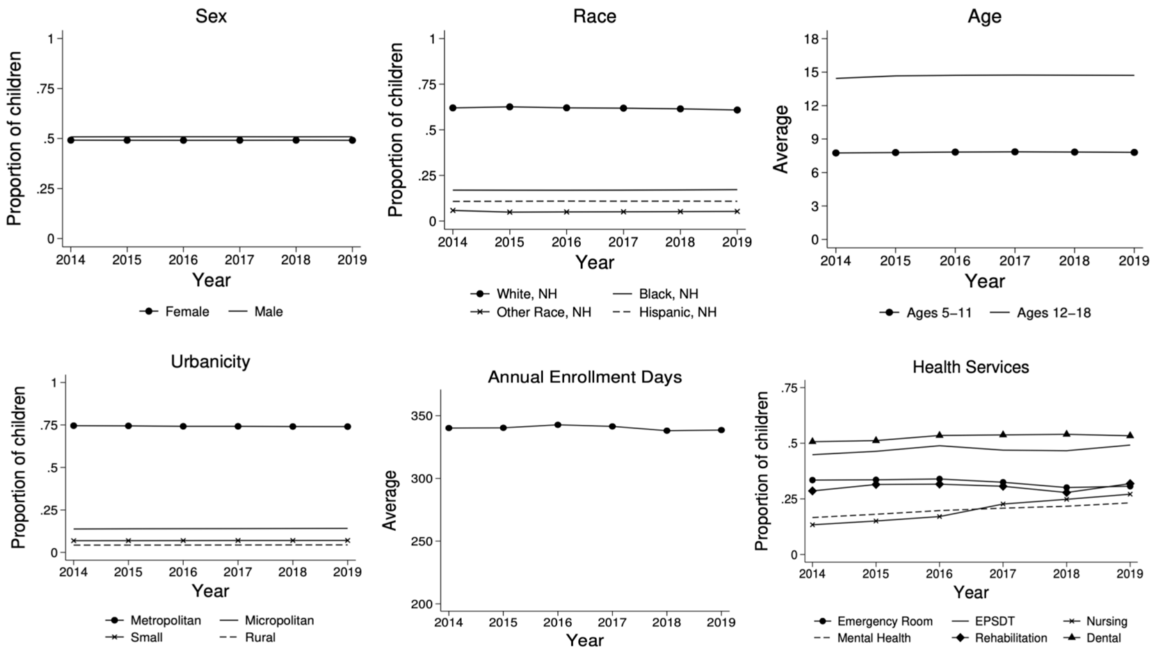

© 2025 Meinhofer A et al. *JAMA Health Forum*.

eTable 2. School-Based Health Services Claims

Measure	Variables	Codes
School Setting	Place of Service	03: School
School Provider	Provider Taxonomy (billing and servicing providers)	251300000X: Local Education Agency (LEA)<br>261QS1000X: Clinic/Center (Student Health)<br>101YS0200X: Counselor (School)<br>103TS0200X: Psychologist (School)<br>1041S0200X: Social Worker (School)<br>163WS0200X: Registered Nurse (School)<br>363LS0200X: Nurse Practitioner (School)<br>364SS0200X: Clinical Nurse Specialist (School)
Individualized Education Plan (IEP)	Benefit Type	060: School-Based Services Payment
	Funding Source	D: Education Agency
	Service Type	0039: School-Based Services (IEP)
	Procedure Codes and Modifiers	T1018: School-based IEP services<br>TM: Individualized education program (IEP)<br>TR: School-based IEP services provided outside public school district responsible for student<br>TL: Individualized family service plan (IFSP)

© 2025 Meinhofer A et al. *JAMA Health Forum*.

eMethods B.2 School-Based Health Services (SBHS)

We identified SBHS claims following an algorithm by CMS, which uses a combination of data elements, including place of service, billing and servicing provider taxonomy, benefit type, service category, funding source, and procedure codes (see *eTable 2*). As noted by CMS, in most states (41 states), a combination of place of service and billing and servicing provider taxonomy codes identify 95% or more of Medicaid-funded SBSH claims identified as SBSH by any means. For some states, category of service and specific procedure codes and modifiers identify most SBHS claims. Medicaid does not require states to follow a standard method for reporting SBHS claims, which created challenges for separately identifying the three types of SBHS measures used in this study. Put differently, a claim for a same SBHS may have been recorded under some data elements but not others depending on the state, and these data elements vary in the specificity of SBHS information being captured. Below we describe some limitations of each of the three SBHS measures, as well as workarounds, including robustness checks, an imputation approach, and exclusion criteria.

(1) *School Setting* using place of service indicating school: This was the best and most consistently collected SBHS measure. A few state-years, however, had considerable missing information in the place of service variable or never identified the school as the place of service despite other data elements in eTable 2 indicating the claim was for SBHS. We therefore entirely dropped states with considerable missing information for multiple state-years as noted in A.4, and implemented an imputation approach for states with modest patterns of missingness. Using the state Medicaid provider ID, we assigned claims with missing place of service as being delivered in "school settings" if (a) over 50% of all other claims with non-missing place of service by the billing or servicing Medicaid provider were identified as delivered in "school

© 2025 Meinhofer A et al. *JAMA Health Forum*.

settings" in the year; or (b) the billing or servicing provider had over 1,000 claims (about the median) delivered in "school settings" in the year or in previous years. (c) Using the state Medicaid provider ID, we also assigned claims as being delivered in "school settings," regardless of missingness in place of service, if over 80% of all claims by the billing or servicing Medicaid provider were identified as delivered in "school settings." We excluded state-years in which the imputed SBHS measure was missing for 30% or more of the beneficiaries. In robustness checks (eTable 7), we dropped several states for which place of service was not necessarily missing but for which the school code was never (or almost never) assigned, despite other data elements indicating school claims. We also analyzed non-imputed measures. We found that effects were robust regardless.

(2) *School Provider* using school provider taxonomy codes: Provider taxonomy in Medicaid claims data was missing for some states and years. Even among states reporting provider taxonomies, some never reported certain school provider taxonomies, which might reflect either state differences in reporting, claiming, or availability of those providers. For example, it is possible that school nurses in state A are enrolled under the school taxonomy code 163WS0200X "Registered Nurse (School)," while school nurses in state B use the school taxonomy code 251300000X "Local Education Agency" or are enrolled under the generic taxonomy code 163W00000X "Registered Nurse." Alternatively, there might be zero school nurses enrolled as a Medicaid provider in state B. We addressed some of these limitations in Medicaid claims data by focusing on an aggregate measure of "School Providers" and using information from the Medicaid Annual Provider Files (APR), which reports multiple provider taxonomies associated with a same provider and can contain information that are missing in Medicaid claims data. Using the state Medicaid provider ID and the APR files, we imputed

© 2025 Meinhofer A et al. *JAMA Health Forum*.

claims with missing provider taxonomy in Medicaid data as being delivered by a "school provider" if (a) in the APR, the billing or servicing provider reported a school-related taxonomy when enrolling as a Medicaid provider (e.g. local education agency, student health center, school nurse, etc.) or if (b) over 50% of all other claims with non-missing provider taxonomy by the billing or servicing Medicaid provider were identified as delivered by a "school provider" in the year. We also assigned claims as being delivered by a "school provider," regardless of missing provider taxonomy in Medicaid claims, if (c) over 80% of all claims by the billing or servicing Medicaid provider were identified as delivered by "school providers." We excluded state-years in which the imputed SBHS measure was missing for 30% or more of the beneficiaries. In robustness checks (eTable 7), we dropped several states for which provider taxonomy was not necessarily missing but for which school-related taxonomy codes were never (or almost never) assigned, despite other data elements indicating school claims. We also analyzed non-imputed measures. We found that effects were robust regardless.

(3) *Individualized Education Program* (IEP) using the data elements benefit type, funding source, service type, and procedure codes: This IEP measure was the most difficult to separately identify as for several states the data elements did not flag any or barely any IEP services in all or most years. IEP services in those states, however, may still be captured when using provider taxonomy or place of service variables, but only in combination with non-IEP SBHS services. We excluded state-years in which the SBHS measure was missing for 30% or more of the beneficiaries. In robustness checks (eTable 7), we dropped several states for which the data elements used for generating IEP services were not necessarily missing but for which IEP codes were never (or almost never) assigned, despite other data elements indicating school claims. We found that effects were robust regardless.

© 2025 Meinhofer A et al. *JAMA Health Forum*.

eMethods 3. Effective Dates of School Medicaid Expansions

The "Free Care Rule" was reversed on 12/15/14 at the federal level. Subsequently, some states began to expand their school Medicaid programs to align with and benefit from the new federal Rule. During our study period of 2014-2019, 12 states expanded their school Medicaid programs to allow Medicaid reimbursement for school-based health services beyond those in the Individualized Education Program of a disabled child with IDEA eligibility. All 12 expanding states were included in our sample. There was considerable state variation in the scope of newly eligible services, students, and providers, as well as in effective dates of state school Medicaid expansions. Additionally, some states expanded through state plan amendment (SPA) while others did not need to amend their state plans to expand.

eMethods C.1. State-Specific Effective Dates

eTable 3 reports state-specific effective dates of school Medicaid expansions during our study period, which we used to code the policy variable of interest as described in the manuscript. We also characterized the scope of school Medicaid expansions in these 12 expanding states based on our interviews with state officials and on documentation in state websites and other official sources.

© 2025 Meinhofer A et al. *JAMA Health Forum*.

eTable 3. Effective Dates of School Medicaid Expansions as of 2019

State	Expansion	SPA Approval	School Medicaid Expansion Date
AR	State Policy	n/a	12/2014
CT	SPA	1/2017	1/2017
KY	SPA	11/2019	11/2019
LA	SPA	10/2015	10/2015
MA	SPA	7/2017	7/2019
MI	SPA	8/2019	8/2019
MO	State Policy	n/a	4/2018
NV	SPA	9/2019	9/2019
NH	State Policy	n/a	8/2017
NC	SPA	1/2019	1/2019
SC	State Policy	n/a	1/2016
WA	State Policy	n/a	12/2014

Notes: We used state-specific effective dates of school Medicaid expansions collected by the Healthy Schools Campaign.[13] Additionally, we interviewed officials from several school Medicaid programs, and read relevant documentation in state websites and other official sources to confirm dates in some cases. This definition included states that expanded Medicaid reimbursement for school-based health services beyond an Individualized Education Program (IEP) either (1) through state plan amendment (SPA) to the Centers for Medicare and Medicaid Services (CMS), using the CMS approval date or the subsequent policy date in the handful of states where additional legislation or action was needed for expansion or (2) through state legislative or administrative policy without the need for SPA, using the implementation date. Among states expanding through SPA, CMS' approval letter often included an SPA approval date and a retroactive date. In this case, we used the approval date. Some states expanding through SPA required additional legislation so that approved SPA changes complied with state law before school expansions could be implemented or agreed on a later date of implementation. In this case, we used the later date (e.g. MA, FL). Some states expanded their school Medicaid programs multiple times (e.g. LA). In this case, we used the first expansion date.

© 2025 Meinhofer A et al. *JAMA Health Forum*.

eMethods C.2. Definitions of Plans of Care for School-Based Services

Individualized Education Plan (IEP) – The IEP outlines the educational services and support for a child with a disability in the United States. The IEP is a key part of the Individuals with Disabilities Education Act (IDEA), which requires public schools to provide free and appropriate education to eligible children with disabilities. Since 1988, Medicaid has provided funding for IEP services.

Section 504 Plan (504 Plan) – The 504 Plan ensures that a child who has a disability under Section 504 of the Rehabilitation Act and is attending an elementary or secondary school receives accommodations and supports that will ensure their academic success and access to the learning environment. The disability must substantially limit a major life activity, which includes the child's ability to learn in a general education classroom.

Individual Healthcare Plan (IHP) – The IHP outlines services for students whose healthcare needs require more complex school nursing services, including students with or at risk for physical or mental health needs.

Behavioral Intervention Plan (BIP) – The BIP outlines an improvement plan created for a student based on the outcome of the functional behavior assessment (FBA). The FBA should identify what is maintaining or causing a challenging behavior, and the BIP specifies the actions to take to improve or replace the behavior.

Individualized Treatment Plan (ITP) – The ITP outlines long-term goals, short-term objectives as well as the recommended scope, frequency, and duration of treatment. The IEP may suffice as the treatment plan if the IEP contains the required elements for an ITP.

Individualized Plan of Care (IPOC) – The IPOC is an individualized comprehensive plan of care to improve the student's condition. The IPOC outlines the presenting problem(s), psychiatric diagnosis(es), goals and objectives, specific interventions, specific services, frequency of services, criteria for achievement of goals and objectives, target dates, and discharge plan.

eMethods C.3. Evidence on Expanding States

In what follows, we characterize the rollout and scope of school Medicaid expansions in the 12 states that expanded during our study period (*eTable 3*). This information is based on our interviews with state officials and review of documentation in state websites and other official sources. We found that some school districts experienced delays when expanding their school Medicaid programs. Most of the states that expanded school Medicaid reimbursement in 2019 and 2020 reported delays due to the COVID-19 pandemic and school closures, therefore we

© 2025 Meinhofer A et al. *JAMA Health Forum*.

dropped 2020 from the sample. Additionally, school district delays in billing school-based health services were associated with training local school staff and difficulties enrolling as a Medicaid provider.[1] However, the extent of these delays likely varied across school districts within a state. For example, some school districts were already enrolled as Medicaid providers prior to 2014 and billed Medicaid for IEP services delivered to children with disabilities and IDEA eligibility. Therefore, it is likely that those school districts were better prepared to hit the ground running and expand their school Medicaid programs to other students.

Arkansas
Expansion (Students): All students
Expansion (Services): Mental health services
State Policy Date of Expansion: December 15, 2014
Source: https://www.medicaid.gov/federal-policy-guidance/downloads/smd-medicaid-payment-for-services-provided-without-charge-free-care.pdf
Information from State Agency: Arkansas expanded services beyond the IEP to include mental health services. Arkansas had made other recent policy changes through their school Medicaid program, Medicaid in the Schools, and was prepared to make this change once the CMS guidance was released on December 15, 2014.

Connecticut
Expansion (Students): Students with 504 Plans
Expansion (Services): 504 Plan services
SPA Date of Expansion: January 10, 2017
Source: https://portal.ct.gov/-/media/departments-and-agencies/dss/health-and-home-care/reimbursement/school-based-healthcare-program/spa_16-0014_-_sch_based_child_health_-_cms_approval_package.pdf?la=en
Information from State Agency: Connecticut expanded services beyond the IEP to include services for students with 504 Plans, but not for all students. This expansion required minimal internal changes as it did not require legislative approval or changes to parental consent procedures because 504 Plan services are under the same special education category as IEP services.

Kentucky
Expansion (Students): All students
Expansion (Services): All medically necessary services
SPA Date of Expansion: November 4, 2019
Source: https://www.medicaid.gov/sites/default/files/State-resource-center/Medicaid-State-Plan-Amendments/Downloads/KY/KY-19-0003.pdf
Information from State Agency: Kentucky expanded services beyond the IEP to include all medically necessary services. In 2019, some schools were able to begin billing for these services,

however, many schools did not begin claiming for these services until the following school year (2020-2021).

Louisiana
Expansion (Students): All students
Expansion (Services): Nursing services
SPA Date of Expansion: October 8, 2015
Source: https://ldh.la.gov/assets/medicaid/StatePlan/Amend2015/15-0019CMS.Approval.pdf; https://healthyschoolscampaign.org/blog/school-medicaid-expansion-publications/
Information from State Agency: Louisiana expanded its school Medicaid program in October 2015 to include school nursing services delivered to all Medicaid-enrolled students. The state's financial analysis showed a 35% increase in federal revenue since the expansion. The program was such a financial success that the state did a second school Medicaid expansion in April 2020 to include all eligible providers and services.

Massachusetts
Expansion (Students): All students
Expansion (Services): Comprehensive but defined benefit package including speech-language pathology, occupational therapy and physical therapy; mental and behavioral health services; skilled nursing services; audiology services; personal care services; medical nutritional counseling; certain physical and behavioral health screenings; fluoride varnish treatment; and Applied Behavior Analysis therapy services for students with an autism spectrum disorder
SPA Date of Expansion: July 17, 2017
Alternative State Date of Expansion: July 1, 2019
Source: https://www.medicaid.gov/sites/default/files/State-resource-center/Medicaid-State-Plan-Amendments/Downloads/MA/MA-16-012.pdf
Information from Agency: Massachusetts expanded services beyond the IEP to include the defined but comprehensive benefits package described above. Massachusetts experienced a delay in SPA implementation from July 17, 2017 to July 1, 2019 due to misalignment between the school-based services covered in the 2017 SPA and the services covered in the Random Moment Time Study which determines how much time eligible providers spend providing services. School districts began expanded billing outside of an IEP starting July 1, 2019, therefore we use this as the effective date.

Michigan
Expansion (Students): All students
Expansion Type (Services); All medically necessary services
SPA Date of Expansion: August 8, 2019
Source: https://www.medicaid.gov/sites/default/files/State-resource-center/Medicaid-State-Plan-Amendments/Downloads/MI/MI-18-0013.pdf; https://healthyschoolscampaign.org/blog/school-medicaid-expansion-publications/
Information from Agency: Michigan expanded services beyond the IEP to include all medically necessary services. Thanks to the comprehensive expansion, the state has seen an increase in reimbursement.

© 2025 Meinhofer A et al. *JAMA Health Forum*.

Missouri
Expansion (Students): All students
Expansion (Services): Behavioral health services only
State Policy Date of Expansion: April 17, 2018
Source: https://mydss.mo.gov/media/pdf/behavioral-health-services-school-setting
Information from State Agency: Missouri expanded services beyond the IEP to include behavioral health services. School-based behavioral health services are billed through two pathways: 1) fee for service when provided to a student who has a disability or medically complex needs or 2) managed care when provided to students without an IEP who do not have a disability or medically complex needs. The Missouri School Board Association (MSBA) Medicaid Consortium has worked since the passage of the policy on April 17, 2018 to establish a relationship with the Medicaid Managed Care Organizations (MCOs) so that non-IEP, behavioral health services delivered to students who do not have a disability or medically complex needs can be billed to Medicaid MCOs.

Nevada
Expansion (Students): All students
Expansion (Services): All medically necessary services
SPA Date of Expansion: October 24, 2019
Source: https://www.medicaid.gov/sites/default/files/State-resource-center/Medicaid-State-Plan-Amendments/Downloads/NV/NV-19-005.pdf
Information from Agency: Nevada expanded services beyond the IEP to include all medically necessary services. The expansion was delayed due to the COVID-19 pandemic disruptions to schools and the revitalization of the expansion began in 2024.

New Hampshire
Expansion (Students): All students
Expansion (Services): All medically necessary services
State Policy Date of Expansion: August 28, 2017
Source: https://legiscan.com/NH/text/SB235/id/1490826
Information from Agency: New Hampshire expanded beyond the IEP to include all medically necessary services. Schools could start billing for non-IEP services as of 2017. In School Year 2019-2020, the Department of Health and Human Services (DHHS) took over the Medicaid to Schools (MTS) program and begun to work toward compliance with the CMS. The final rules for the MTS program have not been published due to ongoing clarification with CMS. Although some New Hampshire schools are billing DHHS for services outside of the IEP, implementation is limited in some areas.

North Carolina
Expansion (Students): Students with a 504 Plan, IHP, or BIP
Expansion (Services): Services within a 504 Plan, IHP, or BIP, including nursing services, psychological and counseling services, occupational, speech and language, audiology, physical therapy services, vision services, and hearing screening services.
SPA Date of Expansion: January 25, 2019
Source: https://www.medicaid.gov/sites/default/files/State-resource-center/Medicaid-State-Plan-Amendments/Downloads/NC/NC-18-0005.pdf

© 2025 Meinhofer A et al. *JAMA Health Forum*.

Information from Agency: North Carolina expanded services beyond the IEP to include 504 Plan, IHP, and BIP services. North Carolina is planning to provide technical assistance to schools to increase non-IEP services billing.

South Carolina
Expansion (Students): Students with an IHP or ITP for rehabilitative therapy services and nursing services and students with behavioral health needs outlined in an ITP or IPOC
Expansion (Services): Rehabilitative services (audiology, physical therapy, occupational therapy, speech and language pathology services) and nursing services within an IHP or ITP and behavioral health services in an ITP or IPOC.
State Policy Date of Expansion: January 1, 2016
Source: https://ed.sc.gov/newsroom/school-district-memoranda-archive/billing-medicaid-and-third-party-liability/scde-free-care-rule-draft-bulletin/
Information from Agency: South Carolina expanded beyond the IEP to include rehabilitative therapy and nursing services within an IHP or ITP and behavioral health services within an ITP or IPOC. Behavioral health services are carved into the Medicaid Managed Care Organization (MCO) plans (billed managed care) and all other services are carved out of the MCO plans (billed fee for service).

Washington
Expansion (Students): All students
Expansion (Services): All medically necessary services
State Policy Date of Expansion: December 15, 2014
Source: https://www.law.cornell.edu/regulations/washington/WAC-182-537-0300
Information from State Agency: Washington expanded beyond the IEP to include all medically necessary services. Since the reversal of Free Care in 2014, local education agencies (LEAs) can contract directly with the Managed Care Organizations (MCOs) to receive reimbursement for providing Medicaid covered health services not included in an IEP. The Health Care Authority did not need to submit a state plan amendment for LEAs contract with the MCOs to receive Medicaid reimbursement for non-IEP services. Although Washington does have a separate section within the state plan for the School Based Health Services program, there is no language throughout the rest of the state plan prohibiting LEAs from receiving Medicaid reimbursement for non-IEP services provided in the school setting. As of May 2023, 7 out of the 9 Educational Service Districts (ESDs) and one school district are licensed behavioral health agencies and are contracted with Medicaid MCOs.

© 2025 Meinhofer A et al. *JAMA Health Forum*.

eMethods 4. Difference-in-Differences Regressions

eTables 4 to 6 report full difference-in-differences (DD) regression output used in Figures 3-5 of the manuscript. We estimated the following DD specification using our longitudinal sample of person-years, where Y_{it} is a binary outcome indexing person *i* in year *t*.

$$Y_{it} = \beta_0 + \beta_1 SME_{st} + \beta_2 X_{it} + \beta_3 Z_{st} + \delta_z + \gamma_t + \varepsilon_{it}$$

The main policy variable SME_{st} was equal to one if state *s* expanded school Medicaid in year *t* and zero otherwise. To minimize measurement error, we assigned SME_{st} to equal the proportion of treated months in the effective year when the effective month was not January. We controlled for individual-level confounders (X_{it}), state-level confounders (Z_{st}), ZIP code fixed-effects (δ_z), and year fixed-effects (γ_t).

© 2025 Meinhofer A et al. *JAMA Health Forum*.

eTable 4. Effect of School Medicaid Expansions on School-Based Health

	Any SBHS	School Setting	IEP	School Provider
Panel A: Ages 5 to 18				
DD	0.089**	0.116***	0.006	0.092**
SE	(0.035)	(0.042)	(0.013)	(0.042)
95% CI	[0.019,0.159]	[0.033,0.200]	[-0.020,0.031]	[0.007,0.177]
P-value	0.014	0.007	0.664	0.034
N	6616250	6520081	5876458	6616250
States	49	49	47	49
Panel B: Ages 5 to 11				
DD	0.090**	0.124***	0.006	0.097**
SE	(0.038)	(0.045)	(0.013)	(0.046)
95% CI	[0.014,0.166]	[0.032,0.215]	[-0.020,0.033]	[0.005,0.190]
P-value	0.022	0.009	0.641	0.04
N	4017014	3955981	3559353	4017014
States	49	49	47	49
Panel C: Ages 12 to 18				
DD	0.088***	0.107***	0.004	0.085**
SE	(0.032)	(0.038)	(0.013)	(0.038)
95% CI	[0.024,0.152]	[0.031,0.183]	[-0.022,0.031]	[0.009,0.160]
P-value	0.008	0.007	0.737	0.029
N	2599236	2564100	2317105	2599236
States	49	49	47	49

Notes: *DD = difference-in-differences estimate, SE = state clustered standard errors, 95% CI = 95% confidence intervals, N = number of person-years in the regression. *** p<0.01, ** p<0.05, * p<0.1*

© 2025 Meinhofer A et al. *JAMA Health Forum*.

eTable 5. Effect of School Medicaid Expansions on Other Health Services by SBHS Claims

	Well-Child Visit	EPSDT	Mental Health	Dental	Rehabilitative	Vision Exam	Hearing Exam	Nursing Services	Non-Emergency Transportation
Panel A: SBSH Claim									
DD	0.007	0.086**	0.017	-0.009	0.024	0.043	0.045	0.074**	-0.008**
SE	0.005	0.04	0.011	0.008	0.025	0.029	0.029	0.031	0.004
95% CI	[-0.003,0.016]	[0.005,0.167]	[-0.006,0.040]	[-0.025,0.006]	[-0.025,0.074]	[-0.016,0.102]	[-0.013,0.103]	[0.011,0.137]	[-0.015,-0.000]
P-value	0.158	0.039	0.14	0.216	0.331	0.152	0.124	0.022	0.045
N	6616250	6616250	6616250	6616250	6616250	6616250	6616250	6616250	6616250
States	49	49	49	49	49	49	49	49	49
Panel B: Any Claim									
DD	0.011	0.041	0.023**	-0.006	0.024	0.025	0.015	0.106*	-0.015
SE	(0.021)	(0.031)	(0.011)	(0.031)	(0.019)	(0.017)	(0.018)	(0.058)	(0.014)
95% CI	[-0.031,0.053]	[-0.021,0.103]	[0.001,0.044]	[-0.068,0.056]	[-0.015,0.063]	[-0.008,0.058]	[-0.021,0.051]	[-0.011,0.223]	[-0.044,0.014]
P-value	0.603	0.193	0.038	0.842	0.222	0.138	0.415	0.074	0.293
N	6616250	6616250	6616250	6616250	6616250	6616250	6616250	6616250	6616250
States	49	49	49	49	49	49	49	49	49

Notes: *DD = difference-in-differences estimate, SE = state clustered standard errors, 95% CI = 95% confidence intervals, N = number of person-years in the regression. *** p<0.01, ** p<0.05, * p<0.1*

© 2025 Meinhofer A et al. *JAMA Health Forum*.

eTable 6. Effect of School Medicaid Expansions on Hospital Services

	Inpatient Stay	Emergency Room
Panel A: Ages 5 to 18		
DD	0.00	-0.013*
SE	(0.005)	(0.007)
95% CI	[-0.010,0.010]	[-0.027,0.001]
P-value	0.961	0.075
N	6616250	6616250
States	49	49
Panel B: Ages 5 to 11		
DD	0.00	-0.018**
SE	(0.005)	(0.007)
95% CI	[-0.010,0.010]	[-0.033,-0.003]
P-value	0.964	0.021
N	4017014	4017014
States	49	49
Panel C: Ages 12 to 18		
DD	0.00	-0.004
SE	(0.006)	(0.007)
95% CI	[-0.011,0.012]	[-0.019,0.010]
P-value	0.966	0.536
N	2599236	2599236
States	49	49

Notes: *DD = difference-in-differences estimate, SE = state clustered standard errors, 95% CI = 95% confidence intervals, N = number of person-years in the regression. *** p<0.01, ** p<0.05, * p<0.1*

© 2025 Meinhofer A et al. *JAMA Health Forum*.

eMethods 5. Sensitivity Analyses

We conducted various sensitivity checks in eTable 7 and eTable 8. In eTable 7, Column (1) reports main estimates for easier comparison; Column (2) excludes all demographic and Medicaid controls; Column (3) replaces ZIP fixed-effects with state fixed-effects; Column (4) replaces ZIP fixed-effects with beneficiary fixed-effects; Column (5) analyzes non-imputed SBHS measures; Column (6) excludes states where data element(s) used to generate a given SBHS measure were present but never (or almost never) indicated school-related codes, despite other data elements indicating school-related codes; Column (7) excludes WA which expanded school Medicaid through Managed Care; and Column (8) employs the novel multiperiod DD estimator, which is robust to bias from treatment effect heterogeneity across states and over time.

In eTable 8, Column (1) excludes four states with low CaseID linkage rates (CT, WA, TX, NC); Column (2) only keeps beneficiaries meeting selection criteria (2) or (3) requiring linkage to O-relatives (see eMethods A), that is, a likely sibling with POUD or a likely parent with OUD; Column (3) only keeps beneficiaries meeting selection criteria (3) requiring linkage to O-parents (see eMethods A), that is, a likely parent with OUD; Column (4) only keeps person-years ≥ first year of POUD exposure ever documented in Medicaid claims to ensure that POUD exposure precedes healthcare use; Column (5) only keeps beneficiaries for which POUD exposure was documented in Medicaid claims for at least two or more calendar years; Column (6) only keeps beneficiaries for which POUD exposure was documented in Medicaid claims for at least four or more calendar years.

© 2025 Meinhofer A et al. *JAMA Health Forum*.

eTable 7. Effect of School Medicaid Expansions on School-Based Health Services, Robustness Checks

	(1)	(2)	(3)	(4)	(5)	(6)	(7)	(8)
	Baseline	No controls	State F.E.	Person F.E.	No imputation	No SBHS reporting	Drop WA	Multiperiod DD Estimator
Panel A: Any SBHS								
DD	0.089**	0.102**	0.082**	0.080**	0.072*	0.092**	0.091**	0.116*
SE	0.035	0.044	0.035	0.037	0.038	0.034	0.036	0.07
95% CI	[0.019,0.159]	[0.014,0.191]	[0.012,0.152]	[0.007,0.154]	[-0.005,0.149]	[0.023,0.160]	[0.019,0.164]	[-0.020,0.253]
P-value	0.014	0.025	0.023	0.033	0.065	0.01	0.015	
N	6616250	6616250	6628404	6628404	6616250	6607623	6591096	6616250
States	49	49	49	49	49	49	48	49
Panel B: School Setting								
DD	0.116***	0.122***	0.111**	0.105**	0.106**	0.120***	0.121***	0.125**
SE	0.042	0.045	0.042	0.045	0.041	0.041	0.043	0.055
95% CI	[0.033,0.200]	[0.032,0.213]	[0.026,0.196]	[0.015,0.195]	[0.023,0.189]	[0.038,0.201]	[0.034,0.208]	[0.018,0.233]
P-value	0.007	0.009	0.011	0.023	0.013	0.005	0.007	
N	6520081	6520081	6532072	6532072	6518652	6096593	6495026	6520081
States	49	49	49	49	49	46	48	49
Panel C: School Provider								
DD	0.092**	0.107*	0.085**	0.090**	0.076**	0.098**	0.095**	0.125*
SE	0.042	0.054	0.041	0.043	0.035	0.044	0.044	0.065
95% CI	[0.007,0.177]	[-0.001,0.215]	[0.002,0.168]	[0.003,0.177]	[0.005,0.147]	[0.009,0.186]	[0.007,0.183]	[-0.003,0.252]
P-value	0.034	0.053	0.046	0.043	0.035	0.031	0.034	
N	6616250	6616250	6628404	6628404	6616250	5704353	6591096	6616250
States	49	49	49	49	49	42	48	49
Panel D: Individualized Education Program (IEP)								
DD	0.006	0.006	-0.002	0.004	0.007	0.01	0.007	-0.002
SE	0.013	0.013	0.011	0.015	0.015	0.014	0.013	0.013
95% CI	[-0.020,0.031]	[-0.020,0.032]	[-0.025,0.020]	[-0.025,0.034]	[-0.024,0.037]	[-0.018,0.039]	[-0.020,0.033]	[-0.028,0.024]
P-value	0.664	0.639	0.83	0.781	0.652	0.467	0.615	
N	5876458	5876458	5888505	5888505	5991116	4389150	5854176	5876458
States	47	47	47	47	49	35	46	47

Notes: *DD = difference-in-differences estimate, SE = state clustered standard errors, 95% CI = 95% confidence intervals, N = number of person-years in the regression. *** p<0.01, ** p<0.05, * p<0.1*

© 2025 Meinhofer A et al. *JAMA Health Forum*.

eTable 8. Effect of School Medicaid Expansions on School-Based Health Services, POUD Exposure

	(1)	(2)	(3)	(4)	(5)	(6)
	Poor Case ID Linkage	Keep O-Relatives	Keep O-Parents	Present or Past Exposure	2+ Years Exposed	4+ Years Exposed
Panel A: Any SBHS						
DD	0.101**	0.091**	0.093**	0.067**	0.095**	0.093**
SE	0.039	0.035	0.035	0.026	0.036	0.036
95% CI	[0.023,0.179]	[0.021,0.162]	[0.023,0.163]	[0.015,0.120]	[0.023,0.166]	[0.020,0.166]
P-value	0.013	0.012	0.01	0.013	0.011	0.014
N	6500415	6540872	6040254	4170754	3767605	1841951
States	45	49	49	49	49	49
Panel B: School Setting						
DD	0.134***	0.120***	0.123***	0.089**	0.127***	0.129**
SE	0.046	0.042	0.042	0.037	0.045	0.049
95% CI	[0.041,0.226]	[0.035,0.204]	[0.039,0.207]	[0.014,0.163]	[0.037,0.217]	[0.030,0.228]
P-value	0.006	0.006	0.005	0.02	0.006	0.012
N	6404710	6445527	5957714	4110534	3715972	1816475
States	45	49	49	49	49	49
Panel C: School Provider						
DD	0.104**	0.095**	0.097**	0.063**	0.093**	0.090**
SE	0.048	0.043	0.042	0.029	0.04	0.037
95% CI	[0.008,0.200]	[0.009,0.181]	[0.012,0.182]	[0.005,0.121]	[0.012,0.175]	[0.014,0.165]
P-value	0.034	0.032	0.026	0.034	0.025	0.02
N	6500415	6540872	6040254	4170754	3767605	1841951
States	45	49	49	49	49	49
Panel D: Individualized Education Program (IEP)						
DD	0.006	0.006	0.006	0.004	0.004	0.001
SE	0.015	0.013	0.014	0.01	0.014	0.014
95% CI	[-0.024,0.035]	[-0.021,0.032]	[-0.022,0.033]	[-0.017,0.025]	[-0.024,0.031]	[-0.027,0.029]
P-value	0.701	0.659	0.691	0.684	0.793	0.95
N	5767787	5810326	5350984	3862492	3354830	1656941
States	43	47	47	47	47	47

Notes: *DD = difference-in-differences estimate, SE = state clustered standard errors, 95% CI = 95% confidence intervals, N = number of person-years in the regression. *** p<0.01, ** p<0.05, * p<0.1*

© 2025 Meinhofer A et al. *JAMA Health Forum.*
