# Supplementary material for: Early School Medicaid Expansions and Health Services for Children With Parental Opioid Use Disorder
Source: JAMA Health Forum. 2025 Jun 13;6(6):e251288. doi: 10.1001/jamahealthforum.2025.1288 (PMC12166484; doi:10.1001/jamahealthforum.2025.1288)
Supplement: Supplement 3. — Data Sharing Statement [file jamahealthforum-e251288-s003.pdf]

Data Sharing Statement

Meinhofer. Early School Medicaid Expansions and Health Services for Children With Parental Opioid Use Disorder. *JAMA Health Forum*. Published June 13, 2025.
doi:10.1001/jamahealthforum.2025.1288

Data
Data available: No
